# Unraveling the MicroRNA tapestry: exploring the molecular dynamics of locoregional recurrent rectal cancer

**DOI:** 10.3389/fonc.2024.1407217

**Published:** 2024-07-12

**Authors:** N. Helge Meyer, Nika Kotnik, Gaetan Aime Noubissi Nzeteu, Léon C. van Kempen, Mirjam Mastik, Maximilian Bockhorn, Achim Troja

**Affiliations:** ^1^ Department of Human Medicine, School of Medicine and Health Sciences, Klinikum Oldenburg, Carl von Ossietzky Universität Oldenburg and University Hospital for General and Visceral Surgery, Oldenburg, Germany; ^2^ Department of Human Medicine, School of Medicine and Health Sciences, Carl von Ossietzky Universität Oldenburg, Oldenburg, Germany; ^3^ Center for Blistering Diseases, University Medical Center Groningen, University of Groningen, Groningen, Netherlands; ^4^ Department of Pathology and Medical Biology, University Medical Center Groningen, University of Groningen, Groningen, Netherlands; ^5^ Department of Pathology, Antwerp University Hospital, University of Antwerp, Antwerp, Belgium

**Keywords:** miRNA, recurrent colorectal cancer, locoregional colorectal cancer, Let-7, stagespecific expression, NanoString expression profiling

## Abstract

**Introduction:**

Colorectal cancer (CRC) ranks as the third most prevalent malignancy globally, with a concerning rise in incidence among young adults. Despite progress in understanding genetic predispositions and lifestyle risk factors, the intricate molecular mechanisms of CRC demand exploration. MicroRNAs (miRNAs) emerge as key regulators of gene expression and their deregulation in tumor cells play pivotal roles in cancer progression.

**Methods:**

NanoString's nCounter technology was utilized to measure the expression of 827 cancer-related miRNAs in tumor tissue and adjacent non-involved normal colon tissue from five patients with locoregional CRC progression. These expression profiles were then compared to those from the primary colon adenocarcinoma (COAD) cohort in The Cancer Genome Atlas (TCGA).

**Results and discussion:**

Intriguingly, 156 miRNAs showed a contrasting dysregulation pattern in reccurent tumor compared to their expression in the TCGA COAD cohort. This observation implies dynamic alterations in miRNA expression patterns throughout disease progression. Our exploratory study contributes to understanding the regulatory landscape of recurrent CRC, emphasizing the role of miRNAs in disease relapse. Notable findings include the prominence of let-7 miRNA family, dysregulation of key target genes, and dynamic changes in miRNA expression patterns during progression. Univariate Cox proportional hazard models highlighted miRNAs associated with adverse outcomes and potential protective factors. The study underscores the need for more extensive investigations into miRNA dynamics during tumor progression and the value of stage specific biomarkers for prognosis.

## 1 Introduction

Colorectal cancer (CRC) is the third most common malignancy worldwide and the second leading cause of cancer death (1). Beyond genetic predisposition (2), the multifaceted etiology of CRC involves an interplay of risk factors such as dietary habits, age, obesity, and physical inactivity, alongside protective factors like regular physical activity and diets rich in fruits, vegetables, and fiber (3–6). Eighty of CRC are localized at the time of diagnosis and surgical resection remains the only curative option (4). However, the recurrence rate of CRC is between 30 to 50% and local recurrence without distal metastases is the most frequent recurrence (7). The recurrence and survival are most influenced by the stage of the tumor. Unraveling the intricate molecular mechanisms underlying CRC necessitates exploring beyond conventional risk factors, delving into the realm of molecular markers. The incidence of CRC is increasing, particularly in the population under 50 (8, 9). Regular screening remains pivotal (10–12), and novel markers are needed for CRC diagnosis, as well as to predict the response to treatment (4, 7).

MicroRNAs (miRNAs) are short non-coding RNAs, 17-25 nucleotides long, that were discovered in 1993 (13). In the past few decades, they have emerged as crucial regulators of gene expression. MiRNAs regulate the gene expression predominantly through the inhibition or degradation of messenger RNAs (14). MiRNAs are also being investigated as possible biomarkers of various diseases (15–17), as well as cancer (18–20). In cancer, including CRC, miRNAs have significant influence on disease prognosis, metastasis and response to treatment. MiRNAs orchestrate complex networks that govern key cellular processes (21–24).

MicroRNAs (miRNAs) are critically involved in the advancement and metastasis of colorectal cancer (CRC). Notably, miRNAs exhibit a dichotomous nature, with some functioning as oncogenic (e.g., miR21, miR221/222, miR135, miR223) and others as tumor-suppressive (e.g., miR-143/145, miR-203, miR-200 family) agents regulating Wnt/β-catenin and TGF-β signaling in CRC (25).

While elevated levels of miR-221 in tumor tissues have been reported in some studies (26, 27), there was no observed difference in miRNA-221 expression between locoregional recurrent CRC tumor tissue and healthy tissue. Increased miR-221 levels have been associated with poor overall survival and a negative prognosis in cancer (28), and it has also been implicated in affecting the efficacy of chemotherapy (29–32). However, the comprehensive understanding of the role of miRNAs in cancer development and progression, particularly within the specific context of CRC, remains an active area of investigation that necessitates further exploration. Particularly, the literature lacks substantial information regarding the miRNA profile of recurrent CRC.

Our exploratory study provides initial insights into the relationship between miRNA expression and tumor progression by investigating the miRNA profile of locoregional recurrent CRC, comparing it to the healthy surrounding tissue within the same patient. While previous studies have explored miRNA expression in primary and metastatic CRC tumors (33, 34), a focused examination of the distinct miRNA landscape associated with locoregional recurrence is lacking. This investigation might fill a critical gap in current understanding of the molecular intricacies underlying CRC relapse and may pave the way for identifying potential therapeutic targets.

## 2 Materials and methods

### 2.1 Selection of patients and patient material

Samples from cancerous and healthy tissue were obtained from 5 patients with a locoregional recurrent CRC that were analyzed in a previous study (35). The cancerous part was taken from the malignancy, while the healthy tissue was taken from the tumor-free tissue margin of the same specimen. Patient characteristics are summarized in **Table 1**.

**Table 1:** Patient characteristics.

Sex	Age (years)	TNM	Primary cancer	Time to recurrence (month)	Treatment	Chemotherapy	Radiotherapy
m	59	rpT4 pN1a pM1 L1V1 R0	rectal cancer	90	pelvic exenteration	yes	yes
m	61	rpT4 pN0 pM1a R0	rectal cancer	36	pelvic exenteration	yes	yes
f	71	rpT4 pN0 cM0 L0 V0 G3 R0	rectal cancer	30	pelvic exenteration	yes	yes
f	75	not available	rectal cancer	112	pelvic exenteration	yes	yes
m	76	rpT4b pN1a cM0 L1 V0 G2 R0	rectal cancer	7	pelvic exenteration	no	no

### 2.2 RNA isolation

Total RNA from 5 tissue sections (20 µm thick) was isolated using the miRNeasy FFPE Kit (Qiagen, Germany) as previously described (35). Briefly, sections were deparaffinized with Heptane. After incubation with proteinase K and heat inactivated samples were centrifuged for 15 min at 20.000 x g. DNA was digested with DNase I. Lysate was then mixed with 100% ethanol and applied to an RNeasy MiniElute spin column. After washing the column RNA was eluted in 30 µl RNase-free water. Total RNA was quantified using the Qubit RNA high sensitivity assay (ThermoFisher) according to supplier’s instructions.

### 2.3 NanoString miRNA expression profiling

The NanoString nCounter technology (NanoString Technologies, Seattle, WA) was used to quantify miRNAs expression according to supplier’s instructions (MAN-C0009-07). Briefly, 100 ng total RNA was used in the adaptor ligation step and subsequently 10 ng was used for hybridization with miRNA capture probes (humanv3: Catalog number CSO-MIR3-12). Post-hybridization, samples were processed on an nCounter SPRINT platform according to supplier’s instructions (MAN-10017).

### 2.4 Data analysis

Data from NanoString assay were analyzed by Rosalind (Rosalind.bio). Normalization, fold changes and p-values were calculated using default criteria provided by NanoString. After background subtraction using POS_A probe correction factors, normalization was conducted in two stages: positive control normalization and codeset normalization. The codeset normalization factor was determined based on the 100 probes with the highest counts. For both stages, the geometric mean of each probeset is utilized to create a normalization factor. ROSALIND calculates fold changes and p-values for comparisons using the t-test method. P-value adjustment was carried out using the Benjamini-Hochberg method to estimate false discovery rates (FDR). The top targeted gene predictions were analyzed in ROSALIND using the multiMiR R library (36).

Gene set enrichment analysis (GSEA) adapted for microRNAs was performed using miRNA Enrichment Analysis and Annotation Tool (miEAA) 2.1 webserver (37).

MiRNA expression data from The Cancer Genome Atlas (TCGA) were analyzed in R. Data from the TCGA Colon Adenocarcinoma (COAD) project, including clinical data and miRNA expression data of 461 patients with CRC, was downloaded using the TCGAbiolinks R package (38). Differential expression was calculated using the DESeq2 R package after removal of miRNAs with less than 10 reads across all the samples and variance stabilizing transformation (39). P-value adjustment was performed using the Benjamini-Hochberg method of estimating false discovery rates (FDR).

Univariate Cox proportional hazard models and Kaplan-Meier curves were generated using the survival and survminer R packages (40).

## 3 Results

**Figure 1:**
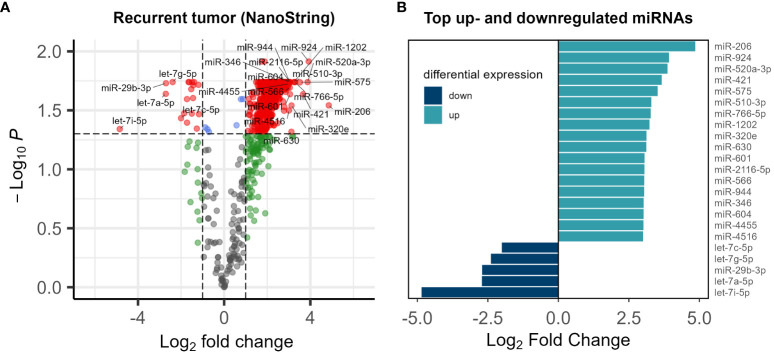
**(A)** Volcano plot of the miRNA expression analysis. Log2 fold change vs. log10 p-value of 798 miRNAs in 5 patients. Significantly dysregulated miRNAs (p-value<0.05) are shown in red **(B)** list of top up- and down- regulated miRNAs Log2 fold change <-2 or >2 and p-value<0.05.

Utilizing NanoString analysis on a comprehensive panel of 827 cancer-related miRNA targets, we successfully detected 798 miRNAs. A comparative analysis of paired normal biopsies from the tumor-free tissue margin and recurrent tumor biopsies from the malignancies of five subjects revealed significant dysregulation in 588 targets (**Figure 1**). MiRNA expression in normal and tumor tissue of the top up- and down- regulated miRNAs (**Figure 1B**) is shown in **Supplementary Figure 1**.

**Figure 2:**
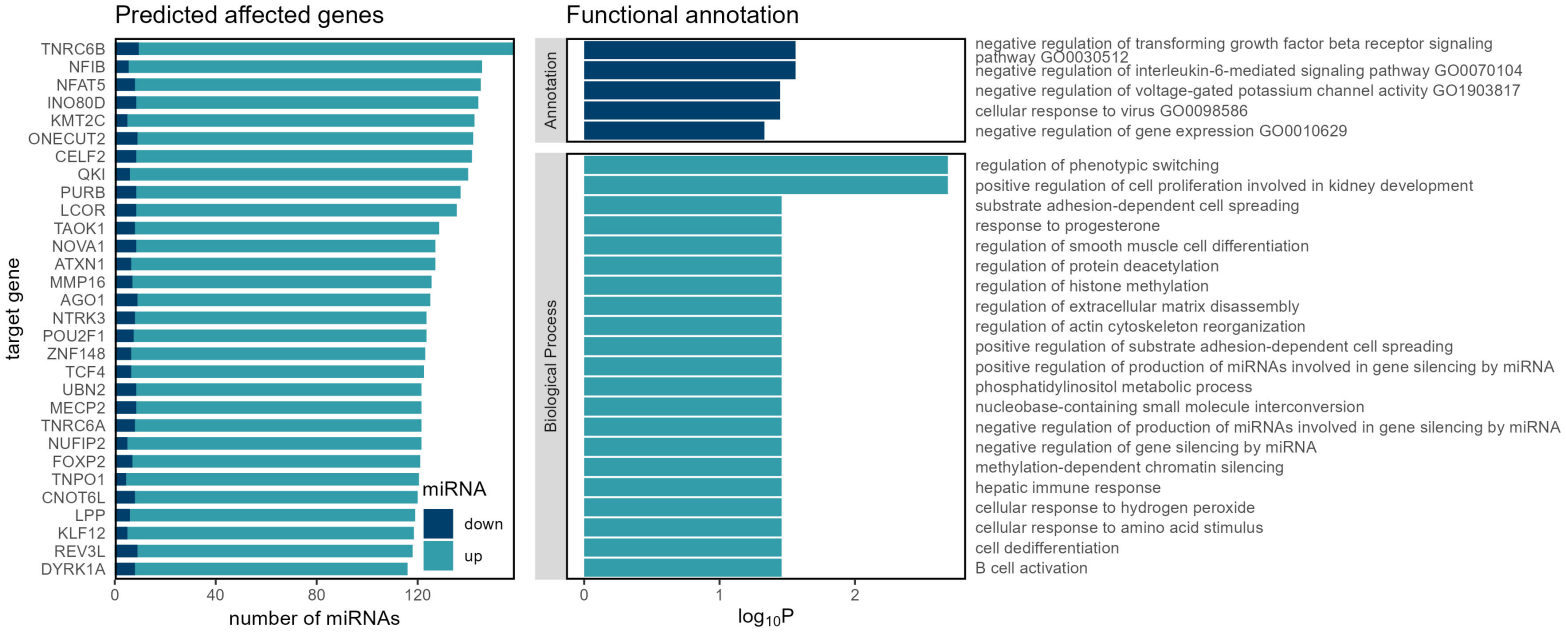
Genes, functions and biological processes that are affected by miRNA dysregulation in recurrent colorectal carcinoma.

Our investigation into CRC relapse identified key target genes, shedding light on potential dysregulation in miRNA processing (**Figure 2**).

The identification of these targets prompted an exploration of enriched biological processes, revealing pathways related to the negative regulation of interleukin-6-mediated signaling, negative regulation of transforming growth factor beta receptor signaling, and cellular response to virus — processes commonly intertwined with immune response and signaling cascades implicated in cancer progression (41, 42).

**Figure 3:**
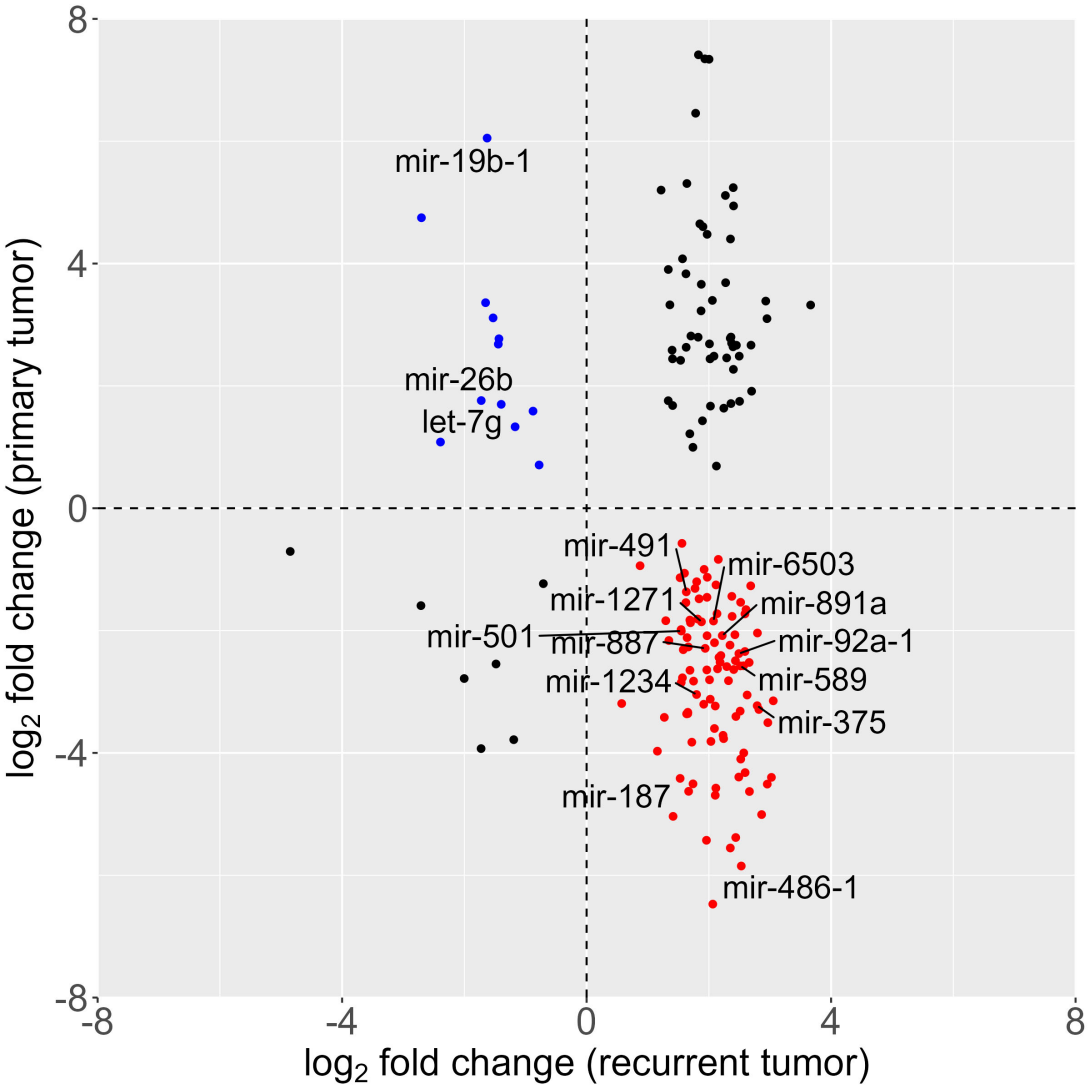
Scatter plot of differential miRNA expression; log2-fold change of tumor vs. healthy tissue in primary (analysis of the colorectal carcinoma cohort, data downloaded from The Cancer Genome Atlas database) and recurrent (NanoString analysis) tumor. (red: overexpressed in recurrent and underexpressed in primary tumor; blue: overexpressed in primary tumor and underexpressed in recurrent tumor).

In our subsequent investigation, we aimed to discern differences in miRNA expression between primary and recurrent tumors. Lacking paired tissue samples from the same patients, we conducted a differential expression analysis using publicly available data from the colon adenocarcinoma (COAD) cohort of The Cancer Genome Atlas (TCGA) database. The dataset included miRNA expression information from 461 patients, encompassing 8 normal tissue samples and 455 primary tumor samples. Due to the scarcity of recurrent and metastatic tumor representation in the cohort (only 1 sample each), these particular samples were excluded from further analysis. The comparative analysis between healthy and primary tumor tissues unveiled dysregulation in 588 out of 1881 detected miRNAs. Remarkably, when comparing our dataset of recurrent tumors with the TCGA dataset of primary tumors, we found an overlap of 777 miRNAs out of the 798 detected in our recurrent tumor dataset. Notably, among the miRNAs that showed dysregulation in both primary and recurrent tumors, 156 exhibited an opposite misregulation pattern., *i.e.* some were upregulated in primary tumors while downregulated in recurrent tumors, or *vice versa*, as illustrated in **Figure 3**. This intriguing observation suggests dynamic changes in miRNA expression patterns during the transition from primary to recurrent tumors, highlighting potential regulatory shifts associated with disease progression.

We then conducted univariate Cox proportional hazard models to assess the correlation between miRNAs with opposite regulation in primary and recurrent tumors and overall survival. The TCGA COAD cohort, lacking data on disease-free progression or time to recurrence, limited our analysis to overall survival as the sole predictor. Out of the 156 miRNAs with opposite dysregulation patterns in primary and recurrent tumors, 14 had statistically significant associations with overall survival, as evidenced by their respective hazard ratios and p-values (**Table 2**).

**Table 2:** Univariate Cox proportional hazard models of miRNAs that show different expression pattern in primary (dataset downloaded from The Cancer Genome Atlas database) and recurrent tumor (NanoString analysis).

miRNA	β-coefficient	Hazard Ratio (95% CI)	p-value
hsa-mir-1234	0.53	1.7 (1.1-2.7)	0.025
hsa-mir-887	0.48	1.6 (1.2-2.1)	0.00081
hsa-mir-26b	0.34	1.4 (1-1.9)	0.03
hsa-mir-6503	0.32	1.4 (1-1.8)	0.03
hsa-mir-491	0.29	1.3 (1-1.8)	0.046
hsa-mir-891a	0.26	1.3 (1.1-1.5)	0.00089
hsa-mir-1271	0.25	1.3 (1-1.6)	0.029
hsa-mir-187	0.22	1.2 (1.1-1.4)	0.004
hsa-mir-615	0.15	1.2 (1-1.3)	0.038
hsa-mir-375	-0.16	0.85 (0.74-0.99)	0.033
hsa-mir-486-1	-0.21	0.81 (0.66-0.98)	0.034
hsa-mir-92a-1	-0.24	0.79 (0.62-0.99)	0.041
hsa-mir-501	-0.3	0.74 (0.58-0.95)	0.018
hsa-mir-589	-0.47	0.63 (0.42-0.95)	0.027

Positive β-coefficient in red, negative β-coefficient in green.

**Figure 4:**
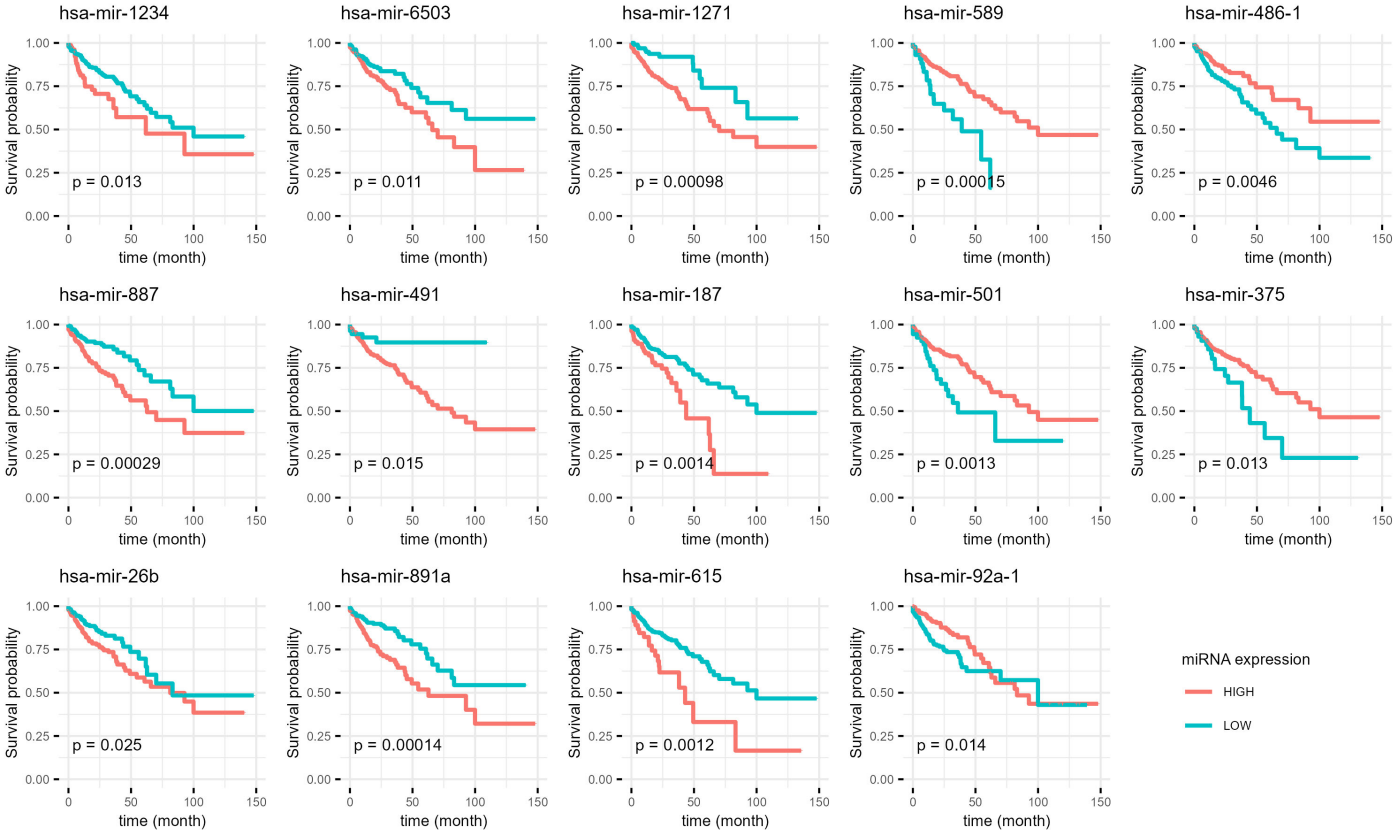
Kaplan-Meier curves of miRNAs which are differentially expressed in primary and recurrent tumor and potential predictors of overall survival. The data from the NanoString analysis of our samples was compared with publicly available dataset from The Cancer Genome Atlas database. P-Value (p) for each is shown for every graph.

The positive β-coefficients and hazard ratios for certain miRNAs (e.g. hsa-mir-1234, hsa-mir-887, hsa-mir-26b) suggest a potential association with increased risk of adverse outcomes. Conversely, miRNAs with negative β-coefficients and hazard ratios below 1 (e.g. hsa-mir-375, hsa-mir-486-1, hsa-mir-92a-1) may indicate a potential protective effect. **Supplementary Figure 2** presents the miRNA expression levels in normal and recurrent tumor tissues, while **Figure 4** displays the corresponding Kaplan-Meier survival curves.

## 4 Discussion

In the broader context of microRNA expression patterns in recurrent CRC, our exploratory study contributes to the growing but still limited understanding of the intricate regulatory landscape associated with disease relapse. MiRNAs play crucial roles in various cellular processes, and their dysregulation has been implicated in cancer progression (43, 44), including CRC (25, 45, 46). In a previous study (35) we focused on the expression of four specific miRNAs — miRNA-21, miRNA-215, miRNA-221, and miRNA-222 — that are implicated in fibrosis and angiogenesis in recurrent CRC (35). A highly variable expression was observed, with only miRNA-21 showing significant upregulation. Our exploratory study of CRC recurrence through miRNA expression analysis has provided insights into the intricate molecular landscape associated with disease recurrence. The dysregulation of 588 miRNAs out of 798 targets (**Figure 1**), with a notable prominence of the let-7 family, aligns with emerging evidence implicating let-7 in CRC development and progression (47–49). Let-7 miRNAs is downregulated in several cancers and is thought to act as a tumor suppressor (47, 50, 51). Notably, miRNAs of the let-7 family emerged as prominently overrepresented among the differentially expressed miRNAs recurrent tumors. This finding provides a nuanced understanding of the miRNA landscape associated with CRC relapse, emphasizing the potential involvement of the let-7 family in the underlying molecular mechanisms of disease recurrence.

Beyond this, the identification of key target genes (**Figure 2**), including *Argonaute RNA-induced silencing complex (RISC) Catalytic Component 1 (AGO1)*, *Trinucleotide Repeat Containing Adaptor 6B (TNRC6B)*, *NOVA Alternative Splicing Regulator 1 (NOVA1)*, and *Matrix Metallopeptidase 16 (MMP16)*, offers valuable clues into potential dysregulation in miRNA processing during cancer relapse (52–55).

*AGO1* is a central player in *RISC*, and both the *TNRC6B* and *NOVA1* are associated with RNA processing. This suggests a link between miRNA dysregulation and the intricate regulatory mechanisms driving cancer relapse (53, 56, 57). These findings echo broader discussions in the field regarding the pivotal role of *AGO* proteins and their interplay with miRNAs in cancer progression (58). The connection with RNA processing factors, like *TNRC6B* and *NOVA1*, further underscores the multifaceted nature of miRNA-mediated regulatory networks in the context of cancer recurrence (57, 59, 60).

*MMP16* is a member of family of proteolytic enzymes and has a high expression in certain tumors (52, 61–63). It is known for its role in extracellular matrix remodeling. Studies link it to processes involved in cancer proliferation and metastasis in several types of cancers (64–66), including CRC (52, 52).

The observed link between these target genes and enriched biological processes associated with miRNA processing highlights the intricate regulatory networks potentially contributing to CRC relapse. Further investigation into *AGO1*, *TNRC6B*, *NOVA1*, *MMP16*, and their connection to miRNA processing could provide deeper insights into the molecular mechanisms underlying cancer recurrence.

Expanding our analysis to the TCGA COAD cohort, we observed an overlap of 777 miRNAs between recurrent tumors and primary tumors, with 156 miRNAs exhibiting opposite dysregulation patterns. This observation aligns with recent studies highlighting the dynamic changes in miRNA expression during cancer progression (43, 67, 68), including in CRC (25). The identification of these miRNAs prompts further investigations into their functional roles and the underlying molecular mechanisms associated with the transition from primary to recurrent tumors (69). MiRNA-19b has been identified as a promising prognostic marker for patient recurrence in CRC in previous studies (70, 71). Suppression of exosomal miR-19b could impact oxaliplatin sensitivity in CRC cell line (72) and miR-19b independently predicted both patient outcome and response to preoperative chemotherapy (70). Interestingly, our current study presents a nuanced perspective, as miRNA-19b was found to be upregulated in the TCGA cohort of primary tumors and downregulated in our cohort of tumor relapse. This discrepancy further underlines the need for further and more extensive studies.

The application of univariate Cox proportional hazard models offered insights into the prognostic significance of miRNAs with distinct expression patterns in primary and recurrent tumors. MiRNAs such as hsa-mir-1234, hsa-mir-887, and hsa-mir-26b, exhibiting positive hazard ratios, align with discussions on miRNA biomarkers associated with adverse outcomes in CRC (26, 73). Conversely, hsa-mir-375, hsa-mir-486-1, and hsa-mir-92a-1, with negative hazard ratios, resonate with studies highlighting miRNAs as potential protective factors in cancer progression (74, 75).

It is essential to acknowledge the limitations of our study, particularly the small sample size (n=5) in an exploratory setting, highlighting the need for more extensive investigations to draw robust conclusions. The use of FFPE samples is a technical limitation of this study. However, it has been shown that NanoString technology is robust and effective even with low-quality RNA samples isolated from FFPE tissue. This technology offers advantages such as sensitivity, reproducibility, and technical robustness in challenging sample conditions (76).

In summary, our study offers preliminary insights into the landscape of recurrent CRC research through integrated miRNA expression analysis, target gene identification, and functional enrichment. While acknowledging the limitations of our small sample size, the dysregulated miRNAs, target genes, and enriched biological processes identified in our study provide valuable groundwork for further investigation. These findings contribute to our evolving understanding of cancer recurrence and offer valuable insights for ongoing discussions in the field. Further investigations into the specific roles of miRNAs, target genes, and the dynamic changes in miRNA expression patterns will be necessary for advancing therapeutic strategies and refining prognostic assessments in CRC.

## Data availability statement

The raw data supporting the conclusions of this article will be made available by the authors, without undue reservation.

## Ethics statement

The studies involving humans were approved by Medizinische Ethikkommision Oldenburg. The studies were conducted in accordance with the local legislation and institutional requirements. The ethics committee/institutional review board waived the requirement of written informed consent for participation from the participants or the participants’ legal guardians/next of kin because this is a retrospective study and some patients had deceased prior to given consent. Data was fully anonymized. Surviving patients have consented to the use of their clinical data and tissue samples.

## Author contributions

NM: Conceptualization, Data curation, Formal analysis, Funding acquisition, Investigation, Project administration, Software, Supervision, Visualization, Writing – original draft, Writing – review & editing. NK: Writing – original draft, Writing – review & editing. GN: Writing – original draft, Writing – review & editing. LV: Investigation, Methodology, Writing – review & editing. MM: Investigation, Writing – review & editing. MB: Funding acquisition, Resources, Supervision, Writing – review & editing. AT: Conceptualization, Funding acquisition, Resources, Supervision, Writing – review & editing.
