# Supplementary material for: Unraveling the MicroRNA tapestry: exploring the molecular dynamics of locoregional recurrent rectal cancer
Source: Front Oncol. 2024 Jul 12;14:1407217. doi: 10.3389/fonc.2024.1407217 (PMC11272531; doi:10.3389/fonc.2024.1407217)
Supplement: Supplementary file 1 [file DataSheet_1.docx]

Unraveling the MicroRNA Tapestry: Exploring the Molecular Dynamics of Locoregional Recurrent Colorectal Cancer

N. Helge Meyer^1,*,†^, Nika Kotnik^2,3,†^, Gaetan Aime Noubissi Nzeteu^1,†^, Léon C. van Kempen^4,5^, Mirjam Mastik^4^, Maximilian Bockhorn^1^ and Achim Troja^1^

^1^Department of Human Medicine, School of Medicine and Health Sciences, Carl von Ossietzky Universität Oldenburg and University Hospital for General and Visceral Surgery, Klinikum Oldenburg, Oldenburg, Germany

^2^Department of Human Medicine, School of Medicine and Health Sciences, Carl von Ossietzky Universität Oldenburg, Oldenburg, Germany

^3^Germany and Center for Blistering Diseases, Department of Dermatology, University Medical Center Groningen, University of Groningen, The Netherlands

^4^Department of Pathology and Medical Biology, University Medical Center Groningen,
University of Groningen, Groningen, The Netherlands

^5^Department of Pathology, Antwerp University Hospital, University of Antwerp, Antwerp, Belgium

†These authors share first authorship.

***Correspondence:**N. Helge Meyer
helge.meyer@uni-oldenburg.de

**
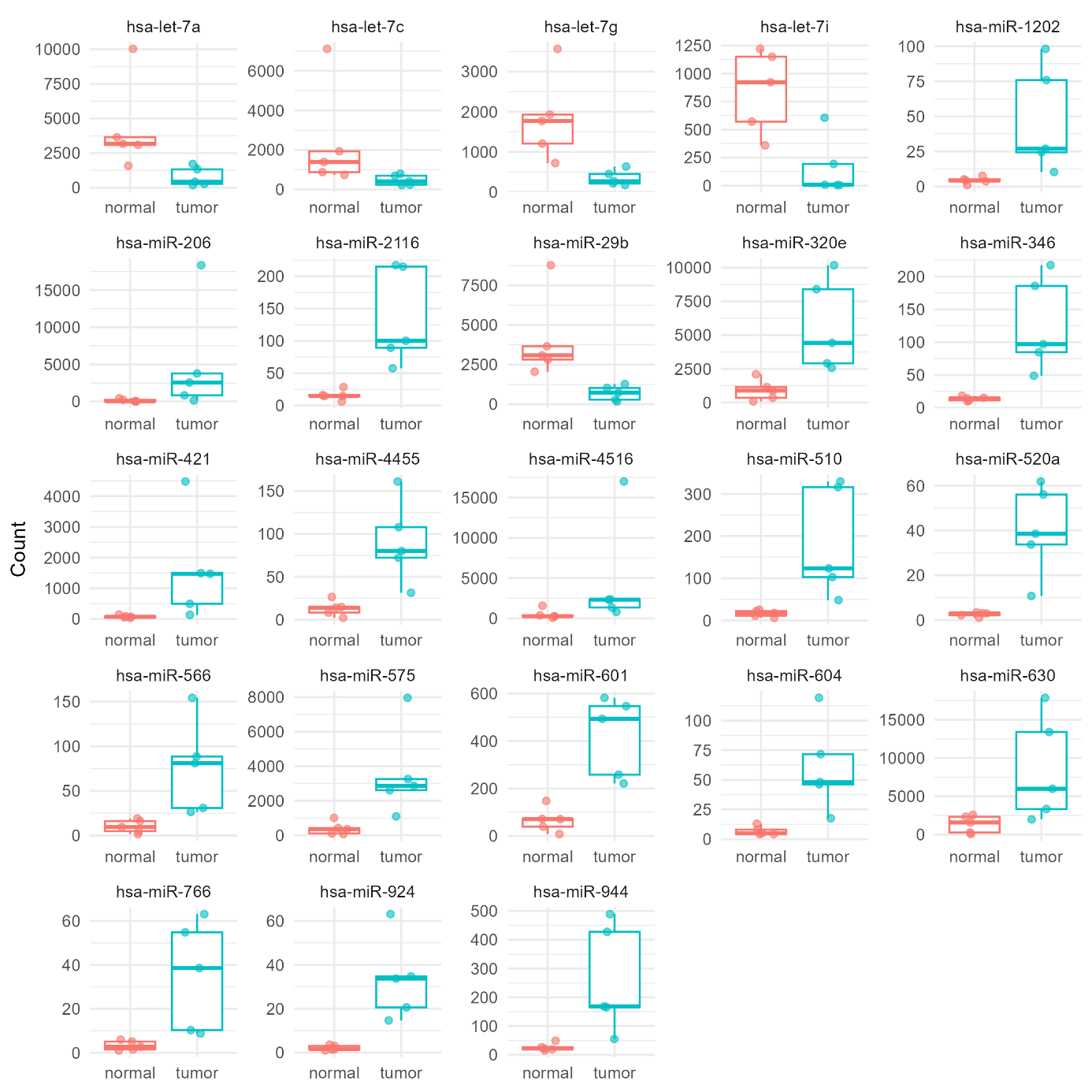
**

**Supplementary Figure 1:** miRNA expression in normal (red) and recurrent tumor (cyan) tissue of top up- and down- regulated miRNAs. Boxplot represents median of normalized counts of the NanoString experiment (line inside the box), interquartile range (box around median) and range of data (whiskers).


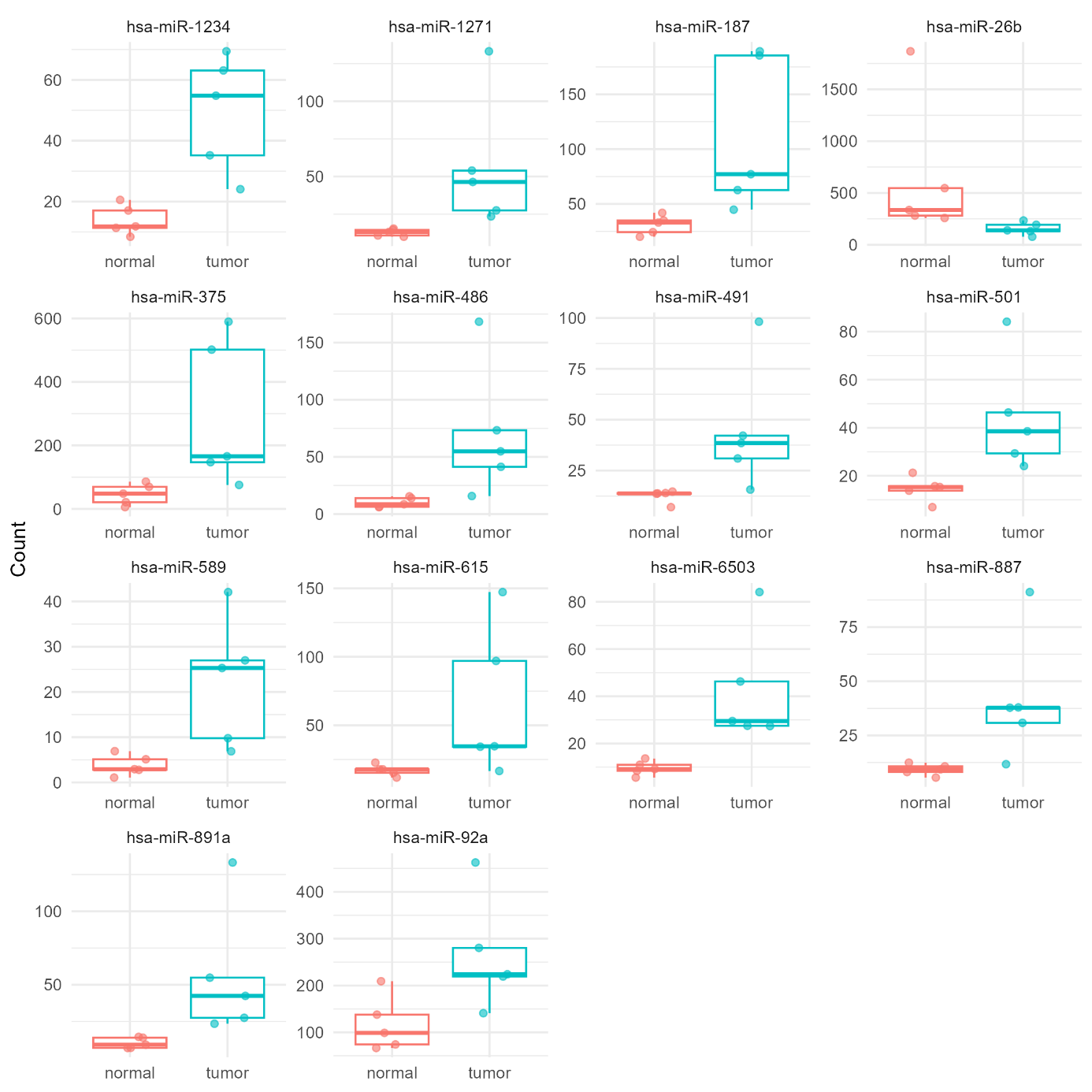


**Supplementary Figure 2:** miRNA expression in normal (red) and recurrent tumor (cyan) tissue of miRNAs which are differentially expressed in primary and recurrent tumor and potential predictors of overall survival (Table 2, Figure 4). Boxplot represents median of normalized counts of the NanoString experiment (line inside the box), interquartile range (box around median) and range of data (whiskers).
